# Analysis of the Relationship Between the Charge Increment of the SARS-CoV-2 Spike Protein and Evolution

**DOI:** 10.3390/v17111483

**Published:** 2025-11-08

**Authors:** Yingxue Ma, Ying Zhang, Menghao Chen, Kun Wang, Jun Lv

**Affiliations:** School of Science, Inner Mongolia University of Technology, Hohhot 010051, China; m18830103730@gmail.com (Y.M.);

**Keywords:** SARS-CoV-2, spike protein, mutation, charge change, evolution

## Abstract

The changes in charge distribution caused by mutations in the spike protein may play a crucial role in balancing infectivity and immune evasion during the evolution of severe acute respiratory syndrome coronavirus 2 (SARS-CoV-2). To explore how charge increments in spike protein variants influence viral evolution, a statistical analysis was conducted on 57 SARS-CoV-2 variants, examining relationships between charge distribution, lineage divergence, angiotensin-converting enzyme 2 (ACE2) affinity, immune evasion, and receptor-binding domain (RBD) expression. A phylogenetic tree was also reconstructed using only the charge properties of mutation sites. Results indicated that with increasing lineage divergence, overall positive charge initially rose sharply and then more gradually. Partitioning the spike protein into three domains—the RBD, the N-terminal flanking region (B-RBD), and the C-terminal flanking region (A-RBD)—revealed distinct patterns: positive charge increased in the RBD and A-RBD, whereas the B-RBD accumulated negative charge. Charge increments were negatively associated with ACE2 affinity and RBD expression but positively correlated with immune evasion. The *k*-mer-based tree derived from charge-reduced sequences showed a topology consistent with the whole-genome tree. These findings suggest that charge distribution in spike proteins is closely linked to viral evolution, with the opposing trends in the RBD and B-RBD potentially reflecting a balance between infectivity and immune escape.

## 1. Introduction

Since its identification in late 2019, severe acute respiratory syndrome coronavirus 2 (SARS-CoV-2) has continued to evolve and spread globally for more than five years. Based on Nextstrain data available as of December 2024, the evolutionary rate of SARS-CoV-2 has been estimated at approximately 29 substitution sites per year [1]. The extensive accumulation of mutations has provided a valuable basis for exploring the evolutionary characteristics of the virus. Among these mutations, those occurring in the spike (S) protein appear to have played a particularly important role in the rapid evolution of SARS-CoV-2 and have therefore become a central focus in studies of viral adaptation [2,3,4,5,6,7]. The S protein represents the principal antigenic component among the structural proteins of SARS-CoV-2. In contrast to other functional proteins, it mediates receptor recognition, cell adhesion, and membrane fusion during the infection process [3]. Viral entry is initiated when the S protein binds to the host angiotensin-converting enzyme 2 (ACE2) receptor [4].

The wildtype S protein exhibits a negatively charged stem region (S2 domain), whereas the tip region (S1 domain), particularly the receptor-binding domain (RBD), is primarily positively charged across a wide pH range [5,6]. During viral evolution, alterations in the charge distribution on the S protein surface have been suggested to influence infectivity and transmissibility [5,6,7,8,9,10,11,12,13,14,15,16,17,18,19,20,21]. Zhang et al. [7] reported that most mutations within the RBD increase surface positive charge or polarity, thereby enhancing binding affinity to ACE2 and facilitating immune evasion. Cotten and Phan proposed that increased positive charge in the S protein may enhance viral transmissibility [8]. Jawad et al. observed that such mutations strengthen electrostatic interactions between the RBD and ACE2, which may contribute to increased infectivity and transmissibility [9]. Lauster et al. demonstrated that the enhanced surface charge of the SARS-CoV-2 spike protein promotes viral adhesion and cellular entry [10]. Nevertheless, in the Omicron variant, the ectodomain (ECD) of the spike protein exhibits no additional increase in surface potential, suggesting a possible evolutionary constraint that balances functional adaptation with structural stability during viral evolution [11]. Božič and Podgornik further noted that, with the emergence of Omicron, the trend toward higher positive charge in SARS-CoV-2 variants was interrupted, and sublineages displayed greater heterogeneity in ionizable amino acid composition [12,13]. Specifically, the spike protein exhibits a progressive increase in the proportion of lysine (LYS) and arginine (ARG) residues [14]. Consistent with this trend, phylogenetic relationships among SARS-CoV-2 lineages can be accurately reconstructed based solely on changes in the count of ionizable amino acids within the spike protein [13]. Uneven charge distribution has also been considered to stabilize the crown-like trimeric structure of the S protein and to promote adhesion of viral particles to negatively charged receptors and membrane surfaces [5], thereby supporting nonspecific electrostatic interactions between the virus and charged macromolecules or extracellular matrices in the surrounding environment [15,16]. Lu et al. [17], analyzing data up to January 2023, reported that positive charge covered nearly the entire interaction surface of the Omicron RBD and Furin cleavage site, which was interpreted as an indication that the potential for further accumulation of positive charges at these interfaces may be limited, possibly constraining additional gains in infectivity.

With the emergence of new variants such as JN.1, KP.2, and XEC, questions remain as to whether the statistical trend of charge accumulation in the S protein will shift, whether regional charge increments will follow distinct evolutionary patterns, and how binding affinity to ACE2 and immune evasion may be influenced by such changes. The present study seeks to address these questions by statistically examining the relationship between lineage divergence and charge increments across different regions of the S protein. In addition, a phylogenetic tree reflecting evolutionary lineages was reconstructed through a simplified reduction in S protein variant sequences. The findings are expected to provide further insight into the evolutionary dynamics of SARS-CoV-2.

## 2. Materials and Methods

### 2.1. Data Collection

The mutation data for the spike (S) protein of 57 SARS-CoV-2 variants were obtained from the Outbreak.info database [22] (https://outbreak.info/, accessed on 15 September 2024). These variants included both early major lineages and more recent subvariants, such as JN.1 and XEC. For each selected variant, information on mutation sites in the S protein was extracted together with the date of the earliest global sampling. Lineage divergence data for the same 57 variants were obtained from Nextstrain [1] (https://nextstrain.org/, accessed on 15 September 2024). Data describing changes in RBD expression levels caused by mutations, as well as their relationship with ACE2 affinity, were derived from the experimental results reported by Starr et al. (2020) [23] (https://jbloomlab.github.io/SARS-CoV-2-RBD_DMS/, accessed on 16 September 2024). Additional experimental data concerning mutation-induced changes in RBD–ACE2 affinity, along with the effects of single amino acid substitutions in the wildtype RBD on immune escape from the antibody LY-CoV1404, were taken from Starr et al. (2022) [24]. Codon-level mutation information for the spike protein coding sequences was obtained from the SARS-CoV-2 CoCoPUT database [25] (https://dnahive.fda.gov/hivecuts/sarscov2/, accessed on 10 December 2024).

### 2.2. SARS-CoV-2 Spike Protein Domains

**Figure 1:**
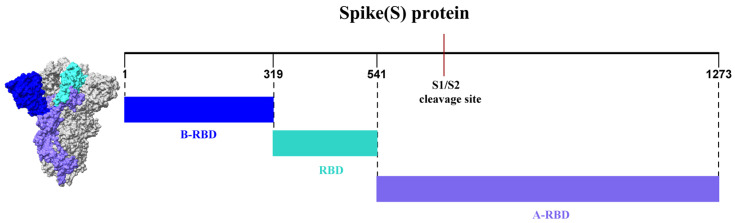
Schematic representation of the regions within the severe acute respiratory syndrome coronavirus 2 (SARS-CoV-2) spike protein. The receptor-binding domain (RBD) spans residues 319–541. The N-terminal flanking region of the RBD (B-RBD, residues 1–318) comprises the signal sequence (residues 1–13), the N-terminal domain (NTD, residues 14–303), and the NTD-to-RBD (N2R) segment (residues 304–318). The C-terminal flanking region of the RBD (A-RBD) extends from residues 542–1273.

The full-length spike (S) protein of SARS-CoV-2 consists of 1273 amino acid residues and is organized into two major domains, S1 and S2. To examine changes in charge increments across different regions of the S protein with increasing lineage divergence, the protein was partitioned into three regions relative to the receptor-binding domain (RBD), as illustrated in Figure 1. Residues 1–318 were designated as the N-terminal flanking region of the RBD (B-RBD), residues 319–541 as the RBD itself, and residues 542–1273 as the C-terminal flanking region of the RBD (A-RBD).

### 2.3. Evolutionary Macro-Lineage Definition of SARS-CoV-2

According to the classification of SARS-CoV-2 variant macro-lineages proposed by Luo and Lv [26], variants predating Omicron (e.g., Alpha, Beta, Gamma, Delta) have been designated as the N-lineage. Early Omicron strains (e.g., BA.1, BA.2, BA.5) have been categorized as the O-lineage, whereas more recently emerging Omicron subvariants (e.g., JN.1, KP.2, XEC) have been grouped into the P-lineage.

### 2.4. Definition of Charge Increment

As suggested by Božič and Podgornik [12,13,14], six amino acids are generally considered ionizable and capable of acquiring charge through protonation or deprotonation. Among them, lysine (LYS, K), arginine (ARG, R), and histidine (HIS, H) are typically classified as positively charged residues, whereas aspartic acid (ASP, D), glutamic acid (GLU, E), and tyrosine (TYR, Y) can bear negative charges. It should be noted that histidine usually carries only a small fractional positive charge at physiological pH, while tyrosine becomes deprotonated and negatively charged only under highly basic conditions. The remaining residues are considered electrically neutral. To quantify charge changes in variants relative to the wild type, a simplified scheme was applied in which positively charged residues were assigned a value of +1, negatively charged residues a value of −1, and neutral residues a value of 0. The charge increment at a single mutation site was defined as the difference between the charge of the mutated residue and that of the wildtype residue. For mutations involving residue deletion, the charge increment for removing a positively charged residue is −1; for removing a negatively charged residue, it is +1; and for removing a neutral residue, it is 0. The charge increment for a designated region is the algebraic sum of the charge increments of all mutated sites within that region. Table 1 presents the Pango lineage names, number of mutation sites, lineage divergence, earliest sampling dates, and charge increments (for B-RBD, RBD, A-RBD, and the full spike protein) of 57 SARS-CoV-2 variants. The number of mutation sites and earliest sampling dates were obtained from Outbreak.info, where lineage-specific mutations are defined as non-synonymous substitutions or deletions present in more than 75% of sequences within a lineage [22]. Lineage divergence was defined as the total number of genomic nucleotide mutations relative to the phylogenetic root [1].

### 2.5. Phylogenetic Tree Construction Scheme

#### 2.5.1. Based on Full-Length S Protein Sequences

An encoding scheme was designed for each variant’s full-length S protein amino acid sequence to reflect mutation status and the resulting charge changes. In this scheme, unmutated sites were encoded as 0, mutated sites with no change in charge as 1, mutated sites with a positive charge increment as 2, and mutated sites with a negative charge increment as 3. In this way, the 20-letter amino acid sequence was reduced to a four-character sequence {0, 1, 2, 3}. It should be noted that the numeric codes (0, 1, 2, 3) are categorical identifiers used solely to distinguish mutation types and do not convey any quantitative weighting of charge magnitude. Accordingly, the resulting distance measure represents mutational dissimilarity rather than relative electrostatic effects.

For the *i*th and *j*th variants, the reduced sequences were denoted as *X_i_* = {*x_i_*_1_, *x_i_*_2_,…, *x_iN_*} and *X_j_* = {*x_j_*_1_, *x_j_*_2_,…, *x_jN_*}, where *i*, *j* ϵ (1, 2,…, *M* = 57). The Euclidean distance between them was calculated as

(1) $$D_{ij}=\sqrt{\sum_{k=1}^{N}{\left(x_{ik}-x_{jk}\right)}^{2}}$$

The complete distance matrix *D* constructed from all reduced sequences was expressed as

(2) $$D=\left(\begin{array}{ccc} D_{11} & \ldots & D_{1M}\\ \vdots & \ddots & \vdots \\ D_{M1} & \ldots & D_{MM} \end{array}\right)$$

Based on this distance matrix, a phylogenetic tree was constructed using the Unweighted Pair Group Method with Arithmetic Mean (UPGMA) [27].

When the reduced sequence itself was directly used as the variant representation, *N* = 1273. Alternatively, when *k*-mer frequencies of the reduced sequence were used, *N* = *w^k^*, where *w* represents the number of character types in the reduced sequence. Since the characters were {0, 1, 2, 3}, *w* = 4. For *k* = 2, this resulted in *N* = 4^2^ = 16. In this case, the 2-mer representation of the reduced sequence was *X* = {*x*_1_, *x*_2_,…, *x_N_*}, where each *x_i_* represented the frequency of a specific 2-mer (e.g., “00,” “01,” “02”) in the reduced sequence.

#### 2.5.2. Based on Mutated Sites of the S Protein

The above tree construction was based on all 1273 amino acid positions of the S protein. Alternatively, a phylogenetic tree was also constructed using only the mutated sites [26]. By taking the union of all mutated sites across the 57 variants, a sequence of *N* = 131 mutated positions was obtained. For each variant, a reduced sequence of length 131 was generated according to the positions of its mutated sites, using the same encoding method described above.

For example, the B.1.1.7 variant contained ten mutated sites at positions {69, 70, 144, 501, 570, 614, 681, 716, 982, 1118}. Applying the encoding scheme, its reduced sequence was represented as {000000000000000031000000000000000000000000000000000000000000000000000000000000000000000000000000000000000000000000000000000000000000000000000000300003002000000000000000000000100000200}. This reduced sequence was then used to construct a phylogenetic tree following the method described in Section 2.5.1.

All statistical computations were performed in R4.5.1 [28]. Cluster analysis was conducted using the hclust (method = “average”) function for the UPGMA method. Phylogenetic trees were visualized using the plot function, and additional figures were generated with ggplot.

## 3. Results

### 3.1. Relationship Between Spike Protein Charge Increment and Lineage Divergence

**Figure 2:**
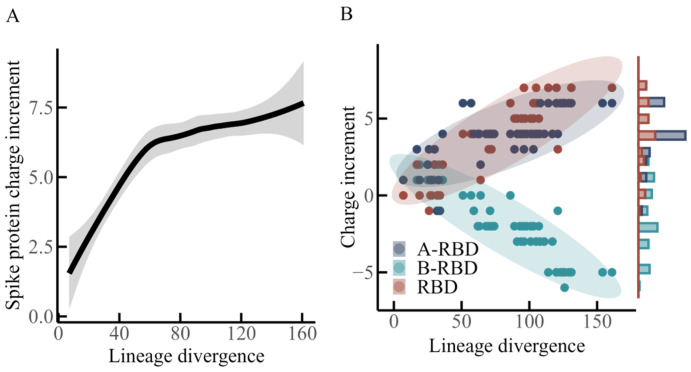
Relationship between spike protein charge increments and lineage divergence. (**A**) Total charge increments of the spike protein in relation to lineage divergence. The trend appears nonlinear: an initial rapid increase is followed by a gradual reduction in growth rate, with a modest rebound at higher divergence levels. The curve was fitted using local polynomial regression, and the shaded area denotes the 95% confidence interval. (**B**) Regional patterns of charge increment across spike protein domains. Within the RBD region, charge increments exhibit a pronounced linear increase with divergence. A comparable but slower upward trend is observed in the A-RBD region. By contrast, the B-RBD region demonstrates a progressive decline in charge increment, shifting toward negative values as divergence increases. Marginal histograms on the right illustrate the distribution of charge increments for each region.

Based on the three defined regions of the spike protein, a statistical analysis was conducted to examine the relationship between charge increments within each region and lineage divergence, as summarized in Figure 2.

Figure 2A illustrates the overall charge increment of the spike protein in relation to lineage divergence. The fitted curve was obtained through local polynomial regression smoothing, with the shaded area indicating the 95% confidence interval. The analysis suggests that within a divergence range of approximately 0–60, the spike protein acquires positive charges in a rapid and nearly linear manner, with an estimated slope of +0.1 charge increment per unit divergence. According to the data in Table 1, this interval corresponds to all N-lineage variants and the early variant of the O-lineage. When divergence extends from about 60 to 120, the rate of positive charge accumulation decreases substantially, with the slope reduced to approximately +0.01. This interval includes BA.2 and subsequent O-lineage variants. A further change appears between divergence values of 120 and 160, where the accumulation of positive charge shows a slight increasing trend again, reflected by a slope of roughly +0.02. This range encompasses all P-lineage variants.

Figure 2B depicts charge increments distributed across the three regions of the spike protein—B-RBD, RBD, and A-RBD—along the axis of lineage divergence. A continuous linear increase in positive charge is observed in the RBD region, accompanied by a comparable, though slightly weaker, upward trend in the A-RBD region. In contrast, the B-RBD region demonstrates a steady decline in net charge with divergence, suggesting a progressive accumulation of negative charges.

Earlier studies [16,17,19] emphasized the global charge increment of the spike protein during evolution, concluding that positive charge increases initially and then tends to stabilize as lineage divergence grows. Although the present dataset incorporates more recent variants (e.g., XEC, first identified in June 2024), the previously reported conclusions appear to remain broadly consistent when viewed at the level of the entire spike protein. However, the region-specific analysis presented here, which differentiates A-RBD, RBD, and B-RBD, highlights an opposing evolutionary trajectory of net charge between RBD and B-RBD (Figure 2B). This pattern resembles the antagonistic trends in net charge evolution between the NTD and RBD regions that were described by Quaranta et al. [29].

### 3.2. Correlation Analysis of Spike Protein Charge Increment, Immune Escape, Affinity, and Expression Levels Across Different Macro-Lineages

**Figure 3:**
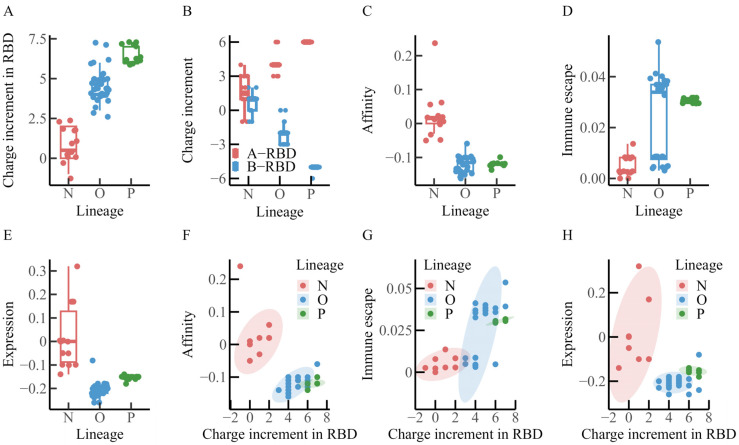
Distribution of charge increments, binding affinity, immune escape, and expression levels of the spike protein across macro-lineages (macro-lineage classification proposed by Luo and Lv [26]). (**A**) Distribution of RBD charge increments. From lineage N to lineage P, the charge increment in the RBD region increases significantly. (**B**) Distribution of charge increments in A-RBD and B-RBD regions. Charge increments in the A-RBD region tend to rise from lineage N to lineage P, whereas those in the B-RBD region appear to decline. (**C**) Distribution of binding affinity. Binding affinity is generally lower in lineages O and P compared with lineage N, suggesting a decreasing trend across evolutionary lineages. (**D**) Distribution of immune evasion capacity. Relative to lineage N, lineages O and P display markedly greater immune evasion capacity. (**E**) Distribution of expression levels. RBD expression levels in lineages O and P are reduced compared with those in lineage N, indicating a potential downward trend during lineage evolution. (**F**) Correlation between binding affinity and RBD charge increment. Overall, the charge increment of the RBD exhibited a negative correlation with ACE2 binding affinity, but specifically for the three lineages, a positive correlation is observed in lineages N and O, whereas no clear association is detected in lineage P. (**G**) Correlation between immune evasion and RBD charge increment. Overall, immune evasion capacity shows a positive correlation with RBD charge increments. (**H**) Correlation between expression level and RBD charge increment. Overall, the charge increment of the RBD exhibited a negative correlation with expression level, but specifically for the three lineages, a positive correlation is apparent in lineage N, while no significant relationship is observed in lineages O and P.

Recent studies have suggested that two major forces may be shaping the evolution of SARS-CoV-2: intrinsic transmissibility, largely determined by ACE2 binding affinity, and immune escape, achieved through reduced susceptibility to neutralizing antibodies [30]. To explore how these evolutionary pressures may be associated with changes in spike protein charge, charge increments in the three regions of the spike protein were examined alongside RBD–ACE2 binding affinity, RBD expression levels, and immune escape across the N, O, and P macro-lineages. In addition, potential relationships among lineage-dependent affinity, immune escape, RBD expression levels, and RBD charge increments were evaluated. The results are summarized in Figure 3 and Table 2.

Charge increments were calculated as described in the Materials and Methods: +1 was assigned to positively charged residues, –1 to negatively charged residues, and 0 to neutral residues. The charge increment per mutation was defined as the difference between the charge of the mutant and that of the corresponding wildtype residue. For each variant, the mean of all corresponding mutation scores, based on the experimental data from Starr et al. [23,24], was calculated to represent its overall affinity, immune escape capacity, and RBD expression level. Specifically, if a variant has *n* mutation sites relative to the wild type, and the affinity increment corresponding to the *i*-th single amino acid mutation is Δ*A_i_*, the overall affinity increment of the variant is given by $\frac{1}{n}\sum_{i=1}^{n}∆A_{i}$. The expression level and immune escape capacity of each variant were calculated in an analogous manner. It should be noted that the data on RBD expression, affinity, and immune escape were obtained using isolated RBD constructs expressed via yeast surface display, as reported by Starr et al. [23,24], rather than from the full-length spike protein.

As illustrated in Figure 3A,B, and summarized in Table 2, charge increments tended to increase in the RBD and A-RBD regions from the N to the P lineage, whereas a decline was observed in the B-RBD region. In addition, within the RBD–ACE2 binding interface, charge increments appeared broadly comparable between the O and P lineages, with values slightly exceeding those observed in the N lineage.

Figure 3C presents the distribution of RBD–ACE2 binding affinity. The N lineage displays a median value of approximately 0.02, whereas the O and P lineages both show medians near −0.12, suggesting a progressive reduction in binding affinity during evolution. Figure 3D illustrates immune escape capacity, which appears to increase gradually across lineages, with median values of approximately 0.0028 for N, 0.034 for O, and 0.031 for P. Among the 32 O-lineage variants, two distinct clusters are observed: one group of 15 earlier Omicron variants (including BA.1, BA.2, and BA.4) exhibits immune escape levels similar to those of the N lineage, whereas the other group of 17 later variants (such as the XBB series and EG.5.1) shows levels closer to those of the P lineage. The similarity between later Omicron variants and the P lineage may reflect a progressive strengthening of immune escape during viral evolution.

Figure 3E shows the distribution of RBD expression levels. The median value is around 0.0 for the N lineage, −0.21 for the O lineage, and −0.15 for the P lineage, suggesting a general decrease in expression levels throughout evolution.

Figure 3F–H further indicate that as the positive charge in the RBD region increases, RBD–ACE2 binding affinity and expression levels tend to decrease, while immune escape capacity increases. Within the N and O lineages, RBD–ACE2 affinity appears to correlate positively with charge increment, although no comparable association is evident in the P lineage. RBD expression levels show a positive correlation with charge increment only within the N lineage, whereas no such relationship is apparent in the O or P lineages. By contrast, immune escape capacity demonstrates a positive correlation with charge increment across all three lineages (N, O, and P).

Taken together, these statistical results suggest that during viral evolution, the RBD region accumulates positive charge, while the B-RBD region (or NTD) accumulates negative charge. At the same time, the affinity between SARS-CoV-2 and ACE2 appears to weaken, whereas immune escape capacity tends to increase. These results are also consistent with the observations of Quaranta et al. [29]. Although correlation does not imply causation, the observed associations between spike protein charge variation and both binding affinity and immune escape may provide insights into the evolutionary mechanisms of SARS-CoV-2.

### 3.3. Analysis of the Correlation Between Codon Usage Characteristics of SARS-CoV-2 Variants and Spike Protein Charge Increment

At the codon level, linear correlations were examined between charge increment and the Codon Adaptation Index (CAI), the Effective Number of Codons (ENC), and GC content (including GC1, GC2, and GC3) across different lineages. The results are presented in Table 3.

From Table 3, it may be observed that, for all variants combined, the charge increment of the spike protein exhibits a positive correlation with ENC (0.49) but negative correlations with GC2 (−0.60) and GC (−0.40), while no meaningful correlation is detected with CAI, GC1, or GC3. Examination of different evolutionary lineages reveals lineage-specific patterns between the charge increment and codon usage of the corresponding genes. In the N lineage, positive correlations are observed with GC1 (0.74) and GC (0.65), whereas negative correlations are found with CAI (−0.56). In the O lineage, a negative correlation is evident with GC2 (−0.50) and GC (−0.49). In the P lineage, negative correlations are present with ENC (−0.62) and GC3 (−0.69).

To conclude, although certain statistical associations are present between codon usage in the S gene and the charge increment of the S protein, these correlations appear generally weak, and changes in several codon-level features during S protein evolution seem to be limited. As noted by Lu et al. [17], such minor changes may not be expected to exert discernible biological effects.

### 3.4. Phylogenetic Tree Construction Based on the Charge Properties of Mutation Sites in the Spike Protein Sequence

Mutations in the spike protein are considered central to the evolutionary dynamics of SARS-CoV-2. Consequently, the reconstruction of phylogenetic relationships among variants might be achieved using only spike protein variant sequences [13]. Luo and Lv [26] proposed a method employing a four-letter sequence to represent amino acid mutations in the spike protein, which was subsequently used to infer phylogenetic relationships through an n-distance algorithm. This approach accounted for the binding affinity between the RBD and ACE2 but did not incorporate the charge properties of mutation sites into the encoding system.

**Figure 4:**
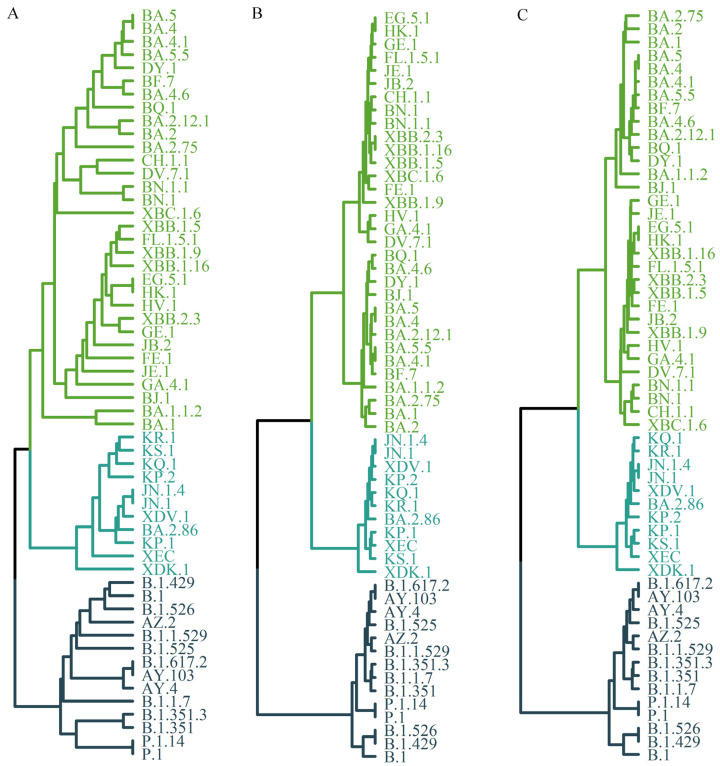
Phylogenetic tree of 57 SARS-CoV-2 variants. (**A**) The phylogenetic tree is constructed from the single-letter sequence of spike protein mutation sites reduced according to charge properties, using the UPGMA method. Three major clades are distinguished, with additional resolution of evolutionary sub-branches such as BA.1, BA.2, and XBB within the O clade, suggesting a relatively fine-grained topological structure. (**B**) Phylogenetic tree based on the 3-mer frequency of the reduced spike protein sequence. The clustering pattern of the major clades appears largely consistent with that shown in panel A, although the internal topology of the O clade is simplified, with fewer resolved evolutionary branches. (**C**) Phylogenetic tree based on the 2-mer frequency of the reduced mutation-site sequence. The overall topological structure of the three clades is retained, while branch details are simplified, preserving the key evolutionary relationships.

In the present study, the amino acid sequence was reduced based solely on the charge characteristics of the mutation sites. The reduced sequences were then encoded using three distinct feature vector construction strategies: (1) direct positional representation of the reduced spike protein sequence with single-letter codes, (2) representation by 3-mer frequencies, and (3) representation by 2-mer frequencies of the reduced mutation-site sequence. The UPGMA algorithm was subsequently applied to construct the phylogenetic tree, and the results are presented in Figure 4.

Figure 4A presents the tree constructed directly from the position-based single-letter representation of the full-length reduced spike protein sequence. The topology is divided into three major branches (highlighted in different colors), which correspond to the three macro-lineages. Within the O lineage, further subdivision into three groups is observed: BA.1-related subtypes, BA.2 and its derivatives (such as BA.2.75 and BA.2.12.1), and the XBB series (e.g., XBB.1.5, XBB.1.9). When the tree is reconstructed using only the reduced sequence of mutation sites, the resulting structure is essentially identical to that in Figure 4A.

Figure 4B illustrates the phylogenetic tree derived from the 3-mer frequency representation of the full-length reduced spike protein sequence, whereas Figure 4C shows the tree constructed from the 2-mer frequency representation of the reduced mutation-site sequence. The major topological framework in both is generally consistent with Figure 4A, with three principal branches corresponding to the macro-lineages. Some differences, however, are evident. In Figure 4B,C, the O lineage is divided into two branches, whereas in Figure 4A, it is separated into three. Moreover, Figure 4A displays more detailed sub-branching, indicating a comparatively more complex structure.

By contrast, the trees in Figure 4B,C appear more simplified, emphasizing the relationships among the primary clades. These differences may be attributed to the distinct approaches used for sequence reduction. While k-mer frequency representation offers a convenient simplification, it may be accompanied by greater information loss.

**Figure 5:**
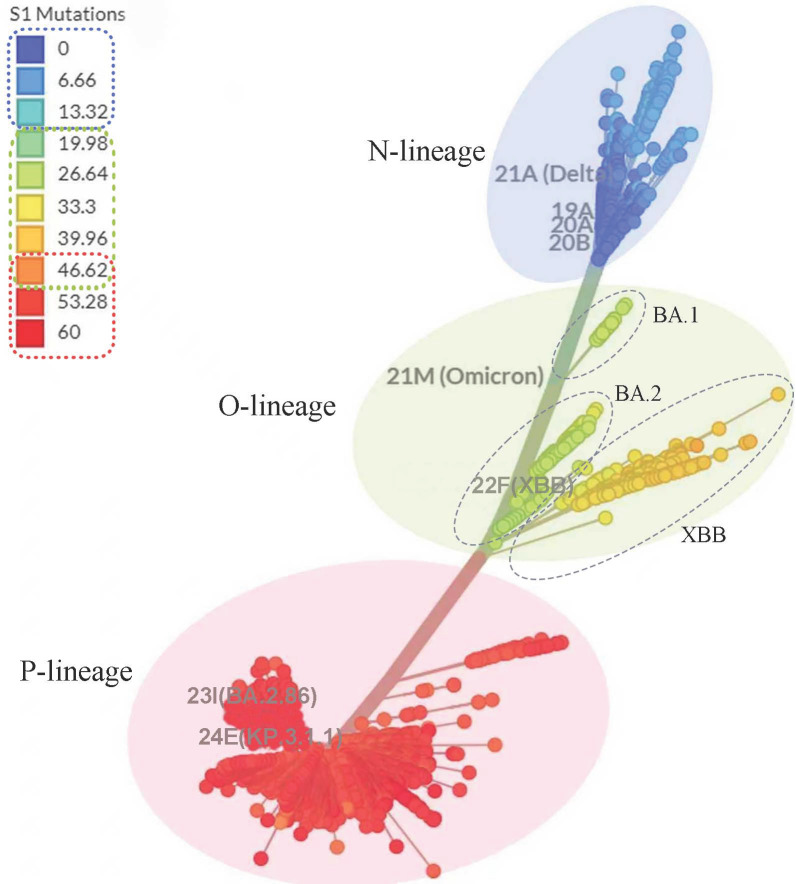
Rootless phylogenetic tree of SARS-CoV-2. The figure illustrates the phylogenetic relationships among 3889 globally representative SARS-CoV-2 variants collected between December 2019 and November 2024. These variants are grouped into three major lineages: N lineage (blue), O lineage (green), and P lineage (red). The O lineage is further divided into three sub-branches: BA.1, BA.2, and XBB. The overall topology of this tree appears broadly consistent with that of the phylogenetic tree constructed from the reduced spike protein sequence shown in Figure 4. The original figure was obtained from Nextstrain (https://nextstrain.org/, accessed on 5 December 2024), licensed under CC-BY-4.0, with minor modifications applied.

For comparison, Figure 5 presents the unrooted phylogenetic tree based on full-genome sequences of SARS-CoV-2 variants obtained from the Nextstrain database [1]. This dataset comprises 3889 globally representative variants sampled between December 2019 and November 2024 (https://nextstrain.org/, accessed on 3 December 2024).

As shown in Figure 5, SARS-CoV-2 variants are clustered into three major branches, which align with the topology observed in the spike-protein–based tree (Figure 4). On closer inspection, the Omicron clade is subdivided into three major groups: 21K (BA.1 and related subtypes), 21L (BA.2 and related subtypes), and 22F (XBB and related subtypes). This subdivision is highly consistent with the branching pattern of the O lineage observed in Figure 4A. Of note, BA.1 forms an early evolutionary branch distinct from other Omicron variants, which may be associated with its 11 unique mutations compared with later sublineages [31]. These mutations may have conferred distinctive evolutionary features that influenced viral transmission and adaptability.

In addition, two mutation-based sequence encoding strategies were applied to reduce the full-length amino acid sequence of the spike protein. In the first strategy, unmutated sites were encoded as 0, mutated sites without charge change as 1, and mutated sites involving charge changes as 2, generating a three-character sequence {0, 1, 2}. In the second strategy, unmutated sites were encoded as 0, and all mutated sites were uniformly encoded as 1, yielding a two-character sequence {0, 1}. Phylogenetic trees reconstructed using these reduced sequences produced topologies largely consistent with those derived from the original sequences. These results suggest that the proposed approach—phylogenetic reconstruction based on charge-informed sequence reduction in the spike protein—may represent a rational and efficient strategy. Furthermore, they imply that charge alterations within the spike protein could contribute to shaping the evolutionary dynamics of SARS-CoV-2 lineages.

## 4. Discussion

### 4.1. Evolutionary Trend of Spike Protein Charge Increment

Previous studies by Cotten and Phan [8], Lauster et al. [11], Božič and Podgornik [12,13], and Lu et al. [17] reported that the overall positive charge of the spike protein tended to increase with lineage divergence. However, this trend appeared to plateau with the emergence of the Omicron lineage.

With the appearance of more recent variants, it remains uncertain whether the charge increment still follows the pattern of “initial increase followed by stabilization.” Analysis of a dataset that includes the latest variants indicates that this trend is maintained from the N to the O lineage. From the O to the P lineage, a slight acceleration in positive charge accumulation is observed; however, the magnitude of change is relatively small and does not appear to alter the underlying pattern of “increasing first and then stabilizing.”

**Figure 6:**
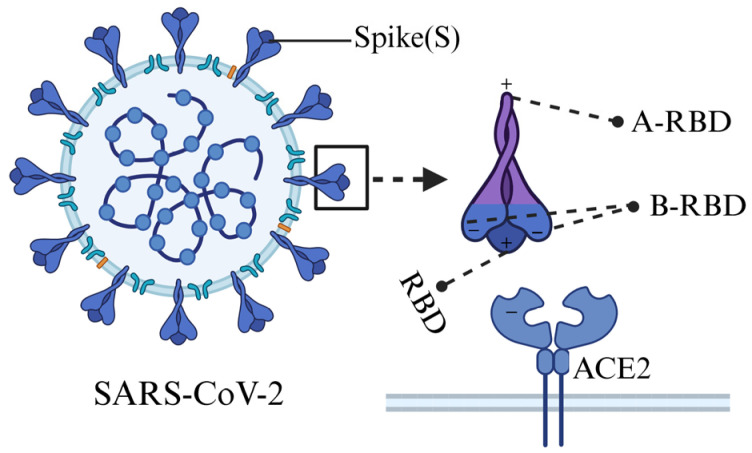
Schematic diagram of the charged structural features of spike protein binding to ACE2. This figure depicts the binding of the SARS-CoV-2 spike protein (S) to the host receptor ACE2 through its receptor-binding domain (RBD). The accumulation of negative charges in the B-RBD region may generate electrostatic repulsion with ACE2, which could partially weaken the affinity between RBD and ACE2. Created with BioRender.com (https://BioRender.com/b1tems8, accessed on 29 March 2025).

A further observation is that the charge increment within the RBD amino-terminal domain (B-RBD) demonstrates an opposite trend compared with that of the RBD itself. Specifically, as the lineages evolve, the positive charge in the RBD increases, whereas the negative charge in the B-RBD region also increases. Figure 6 illustrates the electrostatic features of the spike protein in relation to ACE2 binding. Since the ACE2 binding surface is negatively charged, the accumulation of positive charge within the RBD is expected to enhance electrostatic attraction to ACE2, thereby favoring stronger binding affinity. Conversely, the accumulation of negative charge within the B-RBD region modifies the local surface electrostatic potential and may partially counteract the RBD–ACE2 interaction. This opposite trend in charge increments between the RBD and B-RBD regions may therefore reflect a structural and functional balance between viral infectivity and immune evasion.

### 4.2. Evolution of the Balance Between SARS-CoV-2 Viral Infectivity and Immune Escape

Several studies have suggested that the evolution of SARS-CoV-2 involves a dynamic balance between viral infectivity and immune escape [30,32,33,34,35]. In our analysis, the affinity between the RBD and ACE2 in the O and P lineages appeared markedly lower than that in the N lineage (Figure 3C), whereas immune escape capacity followed an opposite trend (Figure 3D). This observation may imply that SARS-CoV-2 initially favored high infectivity to ensure efficient transmission but, as host immune pressure increased, progressively adopted mutations that compromised infectivity while enhancing immune escape, thereby maintaining an adaptive balance between binding affinity and immune evasion.

To further examine this pattern, RBD expression levels and ACE2 binding changes resulting from additional mutations were also assessed [24]. The results indicated that the relationships among spike protein charge increment, ACE2 affinity, and expression levels generally followed a similar negative correlation to that shown in Figure 3. As early as 2021, Yuan et al. [32] reported that mutations such as K417N and Y505H did not increase ACE2 affinity and even impaired binding, while Xue et al. [33] noted that although epitope-specific mutations in the RBD were central drivers of immune escape, they often incurred an adaptive cost by reducing ACE2 affinity. Nonetheless, compensatory mutations (e.g., N501Y) were shown to partially restore ACE2 binding while remodeling antigenic sites, enabling a balance between immune evasion and receptor engagement. Ma et al. [30] further suggested that during the early stages of viral spread, mutations enhancing ACE2 binding were prevalent and likely contributed to higher transmission efficiency. However, with the rise of population immunity through vaccination and natural infection, selective pressure shifted toward immune escape, with mutations conferring stronger evasion becoming more critical for persistence. Recently, Yang et al. [34] compared BA.2.86 and JN.1 using surface plasmon resonance analysis, reporting that JN.1 exhibited substantially reduced ACE2 affinity but higher immune escape capability. Such a strategy of reduced binding accompanied by enhanced evasion may have allowed these variants to persist at low levels in distinct populations, potentially serving as reservoirs for further accumulation of immune escape mutations [32]. Collectively, these findings suggest that under heightened immune pressure, reduced ACE2 binding affinity may represent one of the adaptive trajectories in SARS-CoV-2 evolution, reflecting the virus’s attempt to balance infectivity and immune evasion.

It is also noteworthy that differences in RBD–ACE2 binding ability and expression levels were observed between the O and P lineages compared with the N lineage, suggesting that evolutionary strategies in these later lineages may rely more heavily on non-charge-related mechanisms. In contrast, the N lineage appeared more sensitive to charge-associated changes. This divergence underscores the possibility of lineage-specific adaptive strategies shaped by distinct evolutionary pressures. Furthermore, weaker RBD–ACE2 binding should not necessarily be equated with reduced transmissibility [34,35], as viral transmission may be influenced by multiple factors across the infection and replication cycle, including structural features of the spike trimer rather than a single RBD. For this reason, despite the lower binding affinity observed in O and P lineages compared with the N lineage, these lineages—particularly O—warrant close monitoring. The P lineage, in particular, may deserve careful attention, as many of its variants exhibit strong immune escape against widely used monoclonal antibodies, potentially raising transmission risks even within vaccinated populations [33].

Beyond charge-altering mutations, the acquisition of new glycosylation sites also plays a crucial role in the evolutionary trade-off [36,37]. The SARS-CoV-2 spike protein is heavily glycosylated, with 22 putative N-glycosylation sites and 17 potential O-glycosylation sites [38]. During viral evolution in human hosts, these sites are often positively selected to enhance glycan shield density, aiding immune evasion and influencing infectivity [36]. A notable example is the N354 glycosylation in the BA.2.86 lineage, which acts as a conformational control element. While it reduces intrinsic infectivity, it provides a selective advantage by facilitating immune escape, illustrating a direct trade-off [37]. Furthermore, epistatic interactions (e.g., N501Y potentiating Q498R) create an “affinity buffer” [39], allowing variants to accommodate immune-evading mutations that might otherwise reduce fitness. Thus, glycosylation works synergistically with other adaptive mutations to balance infectivity and immune evasion [37,39]. However, this paper primarily focuses on how the evolution of SARS-CoV-2 is influenced by charge changes resulting from mutations in the S protein. Although the present analysis does not consider the potential effects of spike protein glycosylation on infectivity and immune escape, it does not alter the main conclusions presented here.

## Figures and Tables

**Table 1:** SARS-CoV-2 variant data.

Macro-Lineage	Variant	NMS ^1^ on Spike Protein	Lineage Divergence	Earliest Date ^2^	Charge Increment			
					B-RBD ^3^	RBD ^4^	A-RBD ^5^	Spike
N-lineage	B.1	1	7	15 January 2020	0	0	1	1
	B.1.617.2	9	17	15 October 2020	1	2	3	6
	B.1.351	10	19	9 July 2020	2	0	1	3
	B.1.525	9	25	11 December 2020	1	2	2	5
	B.1.1.7	10	26	14 May 2020	1	−1	3	3
	B.1.526	4	27	15 November 2020	1	0	1	2
	B.1.429	4	28	6 July 2020	0	1	1	2
	AY.4	10	30	27 October 2020	1	2	3	6
	AY.103	9	30	2 January 2021	1	2	3	6
	P.1	12	32	11 September 2020	−1	0	−1	−2
	B.1.351.3	11	33	4 November 2020	2	0	1	3
	P.1.14	12	34	3 November 2020	−1	0	−1	−2
	AZ.2	6	36	5 February 2021	1	2	4	7
	B.1.1.529	7	64	15 April 2021	0	1	2	3
O-lineage	BA.1	33	51	27 January 2021	0	4	6	10
	BA.1.1.2	37	57	23 November 2021	0	4	6	10
	BA.2	31	59	25 March 2021	−1	4	4	7
	BA.5	34	62	9 December 2021	−2	4	4	6
	BA.5.5	35	63	10 January 2022	−2	4	4	6
	BA.4	34	68	6 January 2022	−2	4	4	6
	BF.7	35	70	24 January 2022	−2	3	4	5
	BA.2.12.1	33	71	28 September 2021	−1	4	4	7
	BA.4.6	36	72	3 January 2022	−2	3	4	5
	BQ.1	36	72	11 January 2022	−2	4	4	6
	DY.1	35	74	25 November 2022	−2	4	4	6
	BA.4.1	35	74	14 December 2021	−2	4	4	6
	BA.2.75	30	86	31 December 2021	0	6	4	10
	CH.1.1	41	89	12 May 2022	−2	5	3	6
	XBB.1.5	42	91	12 June 2022	−3	5	4	6
	BN.1.1	41	93	27 July 2022	−2	4	4	6
	XBB.1.9	40	94	12 October 2022	−3	5	4	6
	XBB.2.3	43	95	21 December 2022	−2	5	4	7
	BJ.1	36	96	15 June 2022	−3	7	3	7
	GE.1	45	97	8 March 2023	−2	5	4	7
	BN.1	40	98	24 January 2022	−2	4	4	6
	FL.1.5.1	44	101	3 January 2023	−3	5	4	6
	XBB.1.16	43	101	4 January 2023	−2	5	4	7
	EG.5.1	44	103	31 January 2023	−2	5	3	6
	HV.1	46	103	29 January 2023	−2	6	4	8
	JB.2	44	105	29 May 2023	−3	6	4	7
	FE.1	41	105	26 January 2023	−3	7	4	8
	HK.1	44	107	12 April 2023	−2	5	4	7
	GA.4.1	47	108	9 May 2023	−3	4	6	7
	JE.1	45	111	8 August 2023	−3	4	4	5
	XBC.1.6	41	121	10 February 2023	−1	3	4	6
	DV.7.1	45	117	29 May 2023	−3	4	4	5
P-lineage	BA.2.86	58	114	11 March 2023	−5	7	6	8
	JN.1	60	120	13 January 2023	−5	7	6	8
	JN.1.4	60	120	20 October 2023	−5	7	6	8
	KQ.1	62	123	10 January 2024	−5	6	6	7
	KP.1	63	125	1 February 2024	−5	6	6	7
	KS.1	64	126	15 February 2024	−6	6	6	6
	KP.2	59	126	2 January 2024	−5	6	6	7
	KR.1	62	129	8 February 2024	−5	6	6	7
	XDK.1	55	131	22 December 2023	−5	7	6	8
	XEC	65	154	28 June 2024	−5	6	6	7
	XDV.1	61	161	26 February 2024	−5	7	6	8

^1^ Number of mutation sites; ^2^ The date of the first sample collection worldwide, data from the outbreak.info website [22]; ^3^ Residues 1–318 of the S protein sequence, designated as the N-terminal flanking region of the RBD (B-RBD); ^4^ Residues 319–541 of the S protein sequence, designated as the receptor-binding domain (RBD); ^5^ Residues 542–1273 of the S protein sequence, designated as the C-terminal flanking region of the RBD (A-RBD).

**Table 2:** The medians of the distribution of charge increment, affinity, immune escape, and expression levels of the S protein in the three macro-lineages.

Variable	Median		
	N-Lineage	O-Lineage	P-Lineage
Charge increment in B-RBD	1.0	−2.0	−5.0
Charge increment in RBD	0.5	4.0	6.0
Charge increment at the RBD-ACE2 binding interface	0.0	1.0	1.0
Charge increment in A-RBD	1.5	4.0	6.0
RBD-ACE2 binding affinity	0.02	−0.12	−0.12
Immune escape	0.0028	0.034	0.031
Expression level of RBD	0.0	−0.21	−0.15

**Table 3:** Pearson correlation coefficient between spike protein charge increment and codon usage features.

Lineage	CAI	ENC	GC1	GC2	GC3	GC
All	−0.04 (0.78)	0.49 (<0.01)	−0.08 (0.58)	−0.60 (<0.01)	0.02 (0.90)	−0.40 (<0.01)
N	−0.56 (0.04)	−0.23 (0.44)	0.74 (<0.01)	0.30 (0.32)	−0.13 (0.68)	0.65 (0.02)
O	0.01 (0.95)	0.10 (0.58)	−0.13 (0.48)	−0.50 (<0.01)	−0.19 (0.27)	−0.49 (<0.01)
P	−0.22 (0.54)	−0.62 (0.05)	0.37 (0.29)	−0.19 (0.59)	−0.69 (0.03)	−0.44 (0.20)

Note: In the table, the value outside the parentheses in each cell denotes the Pearson correlation coefficient, whereas the value inside the parentheses denotes the corresponding *p*-value.

## Data Availability

All data are available in the article text.
